# Characterizing multicity urban traffic conditions using crowdsourced data

**DOI:** 10.1371/journal.pone.0212845

**Published:** 2019-03-12

**Authors:** Divya Jayakumar Nair, Flavien Gilles, Sai Chand, Neeraj Saxena, Vinayak Dixit

**Affiliations:** Research Centre for Integrated Transport Innovation (rCITI), School of Civil and Environmental Engineering, University of New South Wales, Sydney, Australia; University of Washington, UNITED STATES

## Abstract

Road traffic congestion continues to manifest and propagate in cities around the world. The recent technological advancements in intelligent traveler information have a strong influence on the route choice behavior of drivers by enabling them to be more flexible in selecting their routes. Measuring traffic congestion in a city, understanding its spatial dispersion, and investigating whether the congestion patterns are stable (temporally, such as on a day-to-day basis) are critical to developing effective traffic management strategies. In this study, with the help of Google Maps API, we gather traffic speed data of 29 cities across the world over a 40-day period. We present generalized congestion and network stability metrics to compare congestion levels between these cities. We find that (a) traffic congestion is related to macroeconomic characteristics such as per capita income and population density of these cities, (b) congestion patterns are mostly stable on a day-to-day basis, and (c) the rate of spatial dispersion of congestion is smaller in congested cities, i.e. the spatial heterogeneity is less sensitive to increase in delays. This study compares the traffic conditions across global cities on a common datum using crowdsourced data which is becoming readily available for research purposes. This information can potentially assist practitioners to tailor macroscopic network congestion and reliability management policies. The comparison of different cities can also lead to benchmarking and standardization of the policies that have been used to date.

## 1. Introduction

Growing metropolitan cities with strong economies continue to sprawl into suburbs with the desire for private transportation and on-time access to goods and services placing pressure on road networks. Traffic congestion and its repercussions are particularly alarming in developing countries as the existing transport infrastructure struggles to keep up with rapidly increasing productivity [1]. Congestion is characterized by slower speeds, increased travel times, elevated crash rates and rising emissions, placing an economic, social and environmental burden on communities. Over the last few decades, many researchers have provided varying definitions and metrics for traffic congestion that are centered on different traffic characteristics such as vehicle distance traveled [2], travel time [3], delay [4], speed [5,6], volume to capacity ratio [7], level of service [5], and travel cost [8]. Rao and Rao (2012) present an excellent review of the measuring methodologies adopted worldwide [8].

Based on a day-to-day scenario, congestion can be classified into two types: i) recurrent, and ii) non-recurrent. Recurrent congestion is predictable, where the number of users on the road exceeds the road’s design capacity in a sustained manner, such as peak hour. Recent technological advances have influenced the driver’s route choice behavior and have enabled them to be much more flexible in selecting their routes to the destination [9,10]. On the other hand, non-recurrent congestion occurs due to a wide variety of incidents, which can be broadly classified as planned and unplanned incidents. Planned incidents, such as festivals, sporting events, roadworks, and other special events will introduce higher stress on the system than usual. Finally, unplanned incidents are the ones caused by unpredictable events such as vehicle breakdowns, fires, and accidents. Recurrent congestion and planned incidents are likely to be adjusted for by commuters with prior information and experience; however, unplanned incidents are inherently unpredictable, and therefore greatly impact the transport network’s reliability and performance. Furthermore, non-recurrent congestion profoundly impacts the spatial heterogeneity of congestion and network equilibrium. The spatial heterogeneity of congestion, in this context, means that the traffic congestion varies significantly within a city; some locations are congested at a particular instant, whereas other locations are possibly not congested at the same time. The stability of a network in this context refers to the day-to-day stability of the network, which could be affected by weather, incidents, etc. High instability in a transport network results in commuters losing confidence in their expectations of travel time (i.e., decreasing travel time reliability).

A variety of metrics have been used in the past to quantify the impact of traffic congestion in a road network. For example, Long et al. (2008) studied the severity of traffic congestion through traffic propagation and congestion bottleneck identification [11]. Saeedmanesh and Geroliminis studied the spatiotemporal heterogeneity in congested links and observing congestion propagation at the macroscopic level [12]. Calvert and Snelder assess the performance of the road network in terms of its reliability and resilience [13]. Kuang et al. (2013) found an inverse relationship between the reliability of a link/network and the standard deviation of travel time [14].

Understanding the traffic patterns and congestion in multiple cities at once and be able to compare and contrast has been challenging since it is reliant on access to efficient and accurate traffic data. Current standard practice to collect traffic data is by using “in-situ” methods which require physical sensing apparatus such as pneumatic tubes, inductive loops, weigh-in-motion (WIM) sensors, Video Image Processing System (VIPS) and radar detectors [15,16]. Even though physical data collection underpins a majority of transport assessments, the methodologies are expensive to implement and maintain thus limiting network-wide utilization [15,17,18]. Substantial capital outlays are necessary for embedding in-road sensors such as loop detectors which not only involves the purchase and installation of loops but also requires the infrastructure to collect and manage the observed traffic information. Thus, having access to a standard dataset for different cities provides a foundation to assess existing travel conditions. More importantly, such dataset can be used as a benchmark to test different traffic management strategies.

The utilization of crowdsourced traffic data has recently emerged as a viable alternative to the traditional means of traffic data collection. Crowdsourcing involves obtaining information by enlisting or engaging with crowds of people to better understand a process or solve a problem [19]. Crowdsourced transport system data can be collected via diverse data sources, including commercial traffic data providers (Google, Bing, etc.), social media platforms (Facebook, Twitter, etc.), and smartphone navigation apps. Technological developments have resulted in the ubiquitous use of smartphones on a global scale, with over 5 billion people having access to a smartphone [20]. Inbuilt Bluetooth and GPS devices have formed a new option for traffic data collection, referred to as “mobile sensing” [15] and more specifically as “participatory sensing” when related to crowdsourced smartphone data [20–22]. User locations, travel patterns, route selections, travel times and speeds can all be collected using the crowdsourced smartphone data.

Commercial traffic data providers such as TomTom [23], Here [24], INRIX [25], and Google [26] usually offer aggregated historical and real-time traffic information. Nevertheless, only a handful of studies have used crowdsourced data, made available by these providers, to analyze traffic flow and travel time patterns. Tostes et al. (2013) extracted traffic flow information from the Bing Maps Application Programming Interfaces (APIs) to analyze flow density and identify congestion in Chicago [27]. Wang and Xu (2011) estimated the origin-destination (OD) travel time matrix using Google Maps APIs [28]. It can be argued that the proportion of users whose travel data is recorded and processed by these apps is relatively small resulting in a potential bias in representing the real traffic situation.

Nevertheless, these studies (although still only a handful) demonstrate the feasibility of using Maps APIs to reflect traffic patterns that are recorded by the traditional techniques (which, by and large, are considered to be the accurate reflection of the real-world traffic conditions). Furthermore, the volume of data available through these applications grows bigger as the scale of the road network of interest increases which can become a storage issue. However, this shortcoming is rapidly diminishing with the advances in computer technology such as large storage devices (servers) and cloud storage. Lastly, factors such as lack of awareness (about the benefits of crowdsourced data), data privacy and more phone battery consumption among smartphone users can also result in the users avoiding from keeping the location services “on”. While crowdsourced data platforms maintain the confidentiality of collected data by only sharing the aggregated information through the APIs [29], improvement in smartphone technology has resulted in powerful batteries which last longer (in a single charge). Despite all these shortcomings, crowdsourced data has a great potential in complimenting (if not replacing soon) the traditional traffic data collection sources. At this point, it is worth noting that proposing strategies aimed at further improving representation on crowdsourced data and data management is not in the scope of this paper.

In the literature, congestion index is usually defined in terms of traffic parameters such as travel time, traffic speed, traffic flow, etc. where traditional data collection techniques such as loop detectors, pneumatic tubes, sensors, video detection, etc., have served as the primary resources of data. Despite the robust methodologies and meticulous implementation of those congestion matrices, the high costs associated with obtaining the data have proven to be the bottleneck in traffic congestion mitigation and management where most of the rapidly growing cities are looking for monitoring the traffic conditions around the clock. Also, using these congestion metrics to measure congestion, in not a single city but many at once has been a particular challenge in this line of research. In this study, we define a generalized real-time congestion index using just one traffic parameter that can be collected using the crowd-sourced data most cost-effectively. We then analyze the spatial dispersion of traffic congestion in the network and discuss the stability of network equilibrium under various congestion levels and traffic conditions.

## 2. Data collection and preparation

In this study, we gather traffic speed data for 29 major cities around the world for 40 days (between 9^th^ March and 19^th^ April 2017) using Google Maps. Due to the enormous size of these cities, only the road network surrounding the Central Business Districts (CBD) is selected for analysis. Google aggregates speed data from smartphone users to estimate speed and travel times. The data is recorded from devices which use the “*Google Maps*” feature or have their “*My Location*” feature turned on [29]. This data is processed into traffic data using different Application Programming Interface (API), developed by Google, such as distance matrix, directions, speed, and many more. These APIs provide functionality like data analysis, machine learning services (like prediction) or accessing user data (when permission to read the data is given). One of the key outputs from the Google APIs is the traffic speed data. The API calculates a representative speed value from the available crowdsourced data on a road link at any time of the day. Each link is identified by a unique string, called as the “place ID”. A road section is broken down into multiple place IDs at the locations where the road geometry or homogeneity changes (for example, merge and diverge points, intersections, etc.). The APIs provide real-time speed and free-flow speed information for a place ID or a collection of place IDs within a spatial block (based on the tile coordinate system defined by Google). Real-time speed is the speed at which the vehicles are moving on a road segment in the current traffic conditions. Free-flow speed is the typical speed at which a vehicle would be traveling on a road segment in the absence of any traffic. In this study, the link-level speed variation patterns are transformed into a generalized network level metric to compare various cities with different characteristics in terms of congestion index, the rate of spatial dispersion and network stability. Nair et al. (2019) conducted a study to get a clear understanding of the quality of crowdsourced data and how well the data matches the data from traditional sources [30]. The traffic speed data obtained from the Google API is assessed by comparing it with the loop detector traffic speed data for 3 state highways in the city of Portland, Oregon gathered from the Portland Bureau of Transportation and the floating car traffic speed data collected for selected arterial and collector roads in the city of Bandung and Cirebon, Indonesia. The analysis shows that the Google speed data reflects loop detector speed not only at the corridor level but also at a finer spatial resolution of road sections within a corridor. The RMSE value, which quantifies the deviation between the two speed profiles, is around 11 km/h for more than 90 percent of the 53 test locations. The speed comparison between google and floating car data also shows significant temporal similarity, with the difference between the two not exceeding 2 km/h. The results indicate no significant statistical difference between the data sources highlighting the potential of the crowdsourced data as a reliable and cost-effective means of traffic speed data collection.

## 3. Data analysis

In this section, we define a generalized congestion index to compare the traffic profiles of 29 cities. We then examine the impact of traffic congestion on these cities by analyzing the spatial heterogeneity, formation and dissipation and reliability of congestion. We also assess the existence of a stable network equilibrium condition across days.

### 3.1 Congestion index

Traffic congestion refers to the incremental delay experienced by vehicles in the road network, particularly as traffic volume approaches the capacity [31,32]. Congestion Index (CI) is highly useful for making decisions, such as, route choice (how to travel across the cities, particularly during the peak hour), infrastructure expansion, traffic control (coordinated traffic signal system), travel demand management (congestion pricing policies to induce people to change their trip time), etc. [8]. We measure Congestion Index (CI) as the proportion of travel time lost in congestion based on the traffic speed information considering some of the desirable attributes provided by Levinson and Lomax (1996), Boarnet et al. (1998) and Lomax et al. (1997). According to these researchers, a congestion index/metric should (i) be easy to communicate, (ii) measure congestion at network level, (iii) be based on widely available data, (iv) allow comparison across metropolitan cities (v) have statistical analysis capability and (vi) provide a continuous range of values [3,33,34]. Lyman and Bertini (2008) defined CI as the ratio of travel time required during peak traffic periods to the normal travel time [35]. Similarly, Gao et al. (2016) used the ratio of vehicle speeds during peak traffic periods to normal speeds to define CI [36].

In this study, we adopt CI as a relative measure that compares the current traffic speed to the free-flow speed, a threshold value that represents the beginning of the delay. The free-flow speed (*V*_*f*_) is defined as the maximum speed during the off-peak period, that usually occur during the night in most of the cities (this was empirically verified from the collected data). Then the congestion index of a link *i* at time *t* (*CI*_*it*_) is computed as the ratio of its delay ($\delta_{it}=\frac{1}{V_{it}}-\frac{1}{{V_{f}}_{it}}$) and the free flow travel time/km (given in Eq 1), and the CI of the network at time *t* is computed as the average *CI*_*it*_ of all the links in the network within a day.

$$CI_{it}=\frac{\delta_{it}}{\frac{1}{{V_{f}}_{it}}}$$ (1)

**Fig 1:**
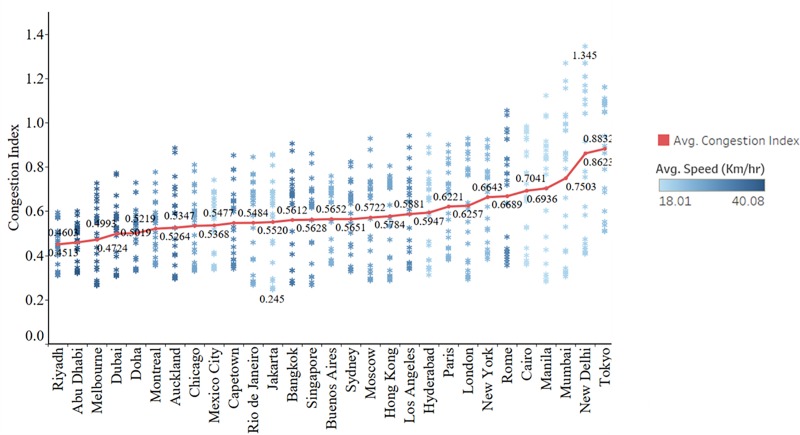
Estimated CI and observed average speed. CI is a relative measure that compares the current traffic speed to the free-flow speed, a threshold value that represents the beginning of the delay. The blue dot points represent the estimated CI for each hour of a day. The red dot points represent the average estimated CI per day. A color gradient is used to show the variation in average speed of the city.

The estimated CI for each hour of a day and the average CI along with the average speed of the cities are presented in Fig 1.

We found that in cities like New Delhi, Mumbai, Tokyo, Cairo and Manila on an average more than 70% of travel time is lost in congestion with a maximum of 135% (New Delhi). Most of them are the cities in developing economies of the world, and all of them are densely populated cities that are expected to have highly congested traffic conditions. All other cities except the ones from the Middle East (Abu Dhabi, Riyadh, Doha, etc.), exhibit moderate congestion with an average CI between 0.5 and 0.7. Although the average congestion is moderate, some cities like Rome, Bangkok, Hong Kong, New York, Los Angeles, London, Hyderabad, and Paris show a high level of congestion (closer to 100%) during the peak hours. Most of them are tourist cities with a high global destination index, which would add extra traffic to the city network. As shown, different cities have different CI that varies across time.

**Fig 2:**
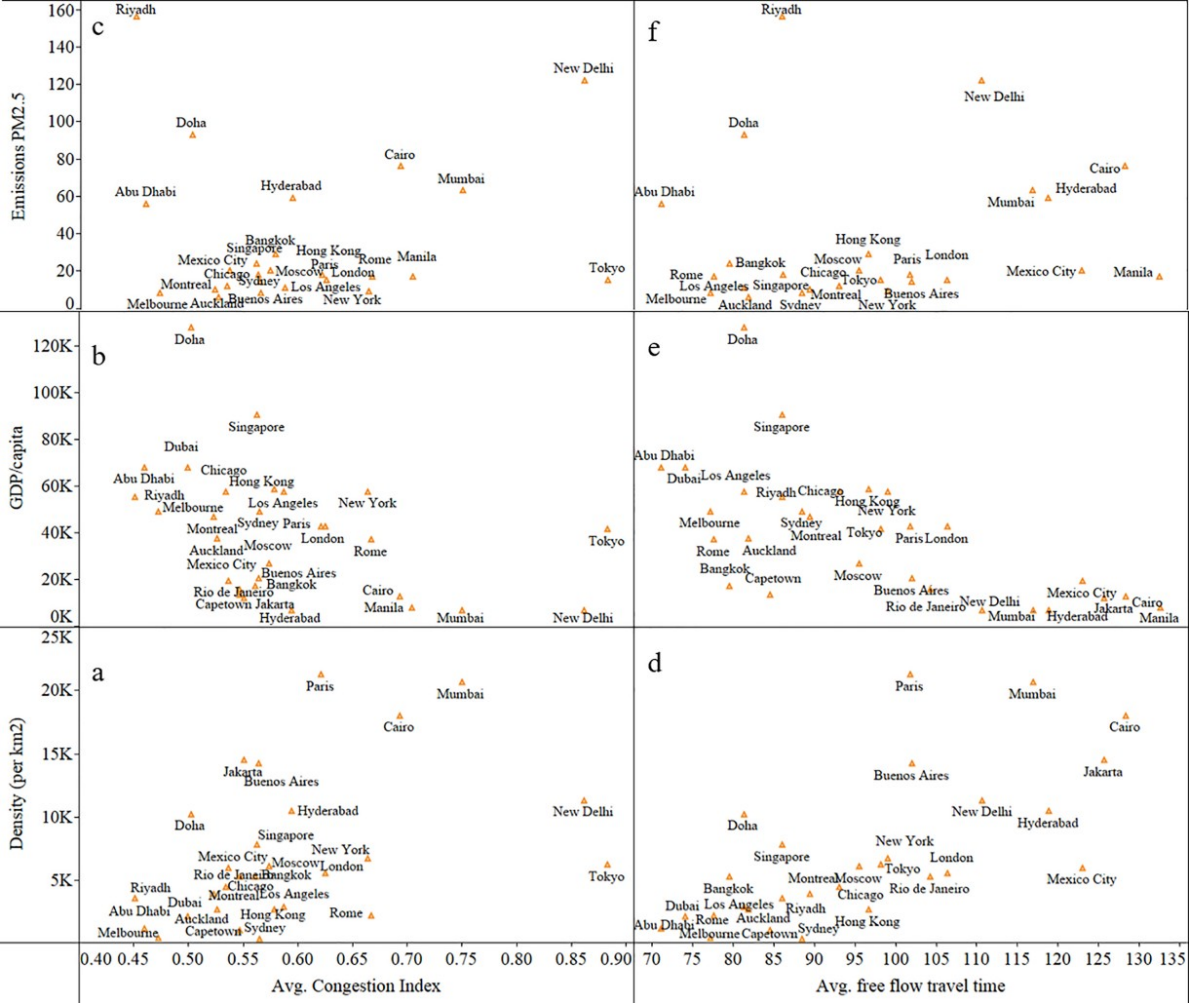
Comparison of cities. **a.-c.** average CI of 29 cities are fitted against the population density, GDP/capita and vehicular emissions respectively and **d.-f.** the free flow speed of 29 cities are fitted against the population density, GDP/capita and vehicular emissions respectively.

Further, the relation between the proposed CI and different macroeconomic features of cities are explored in this study. Fig 2 shows there is a positive correlation between the CI, population density (*r* = 0.68) and emissions (*r* = 0.57). This observation is intuitive because as the number of individuals increases in a small area (for example the CBD of a city), it leads to more usage of automobiles for commute activities. The emissions PM2.5 concentration corresponds to the fine particles (less than 2.5 μm) in a city’s atmosphere. The concentration is lower than 50μm/m^3^ limit given by WHO for the cities in developed economies [37]. The rest of the cities have a higher amount of this quantity which indicate higher motor vehicle usage.

The CI shows a negative relationship with the GDP (*r* = −0.45) per capita of a city. This result is intuitive as well given that the low-income countries (the ones with lower GDP per capita) typically have mixed traffic conditions, encompassing a wide variety of vehicles competing for available road space. Moreover, traffic in such countries is characterized by poor lane keeping behavior among drivers, loose traffic regulations and enforcement, side friction, and complex interactions between vehicles [38,39]. All these factors result in the chaotic nature of the urban road traffic network, further leading to more traffic related incidents and congestion. Nevertheless, Tokyo seems to be a clear outlier with the general trend and hence needs further investigation, which is not within the scope of this study. Furthermore, it is interesting to notice that not only the CI, but the average free-flow travel time is strongly related to the city’s macroeconomic characteristics. These results demonstrate the applicability of the crowdsourced traffic speed dataset and also the proposed CI.

Furthermore, a multivariate regression model is developed to express the congestion index in terms of macroscopic indicators of the city such as population density, GDP per capita and particulate matter emissions. Since, the dependent variable, CI, represents a fraction, a Generalised Linear Model (GLM) is developed for the given dataset in STATA [40]. The selected generalised linear model is a logistic regression (family binomial) with logit link function. Table 1 shows the estimated parameters from the developed model. The table shows the estimated coefficient for population density as 20.5 which implies that an increase in the population density is more likely to cause an increase in the CI. This observation makes sense as a dense population would mean more traffic volume resulting in a greater CI. The parameter for GDP per capita is negative and significant (-4.75) which indicates an inverse relationship with CI. A higher GDP indicates a developed economy which can provide appropriate transport infrastructure to suffice the growing traffic demand. Lastly, the particulate emission ratio is positive and significant (0.884) which indicates a positive relationship to the CI. It has been found that PM2.5 emissions, which is more hazardous than PM10, form almost 75 percent of PM10 [41]. Thus, the ratio PM2.5 by PM10 represents the degree of emissions hazard. An increase in the ratio implies more emissions, probably due to more traffic within a city, which leads to a higher CI. The likelihood value at convergence is found to be -12 and the AIC value of the model is 1.19.

**Table 1:** Estimated parameters from the GLM.

Parameter	Estimated coefficient
Population density	20.5 ***
GDP per capita	-4.75 ***
PM2.5/PM10	0.884 ***
Constant	-0.045
Goodness of fit	
Log-likelihood	-12.0
No. of observations	29

*** significant at 99% confidence interval

### 3.2 A comparison of within-day congestion propagation and dissipation profiles

**Fig 3:**
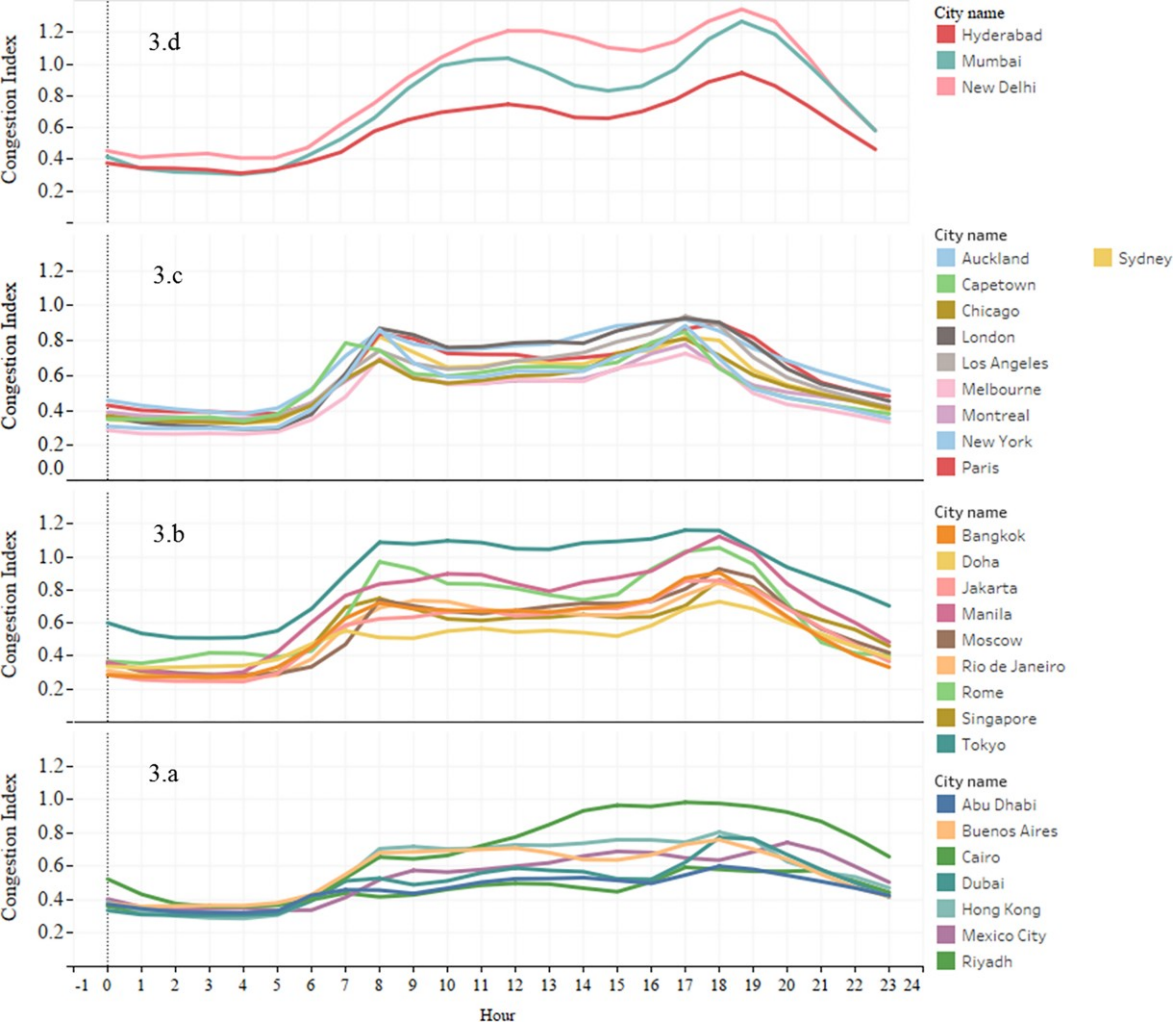
Temporal distribution of congestion index. **a.** cities with a low congestion index, **b. and c.** cities with moderate to high congestion index, **d.** an exception where the rate of dissipation is found to increase as the congestion increases.

**Fig 4:**
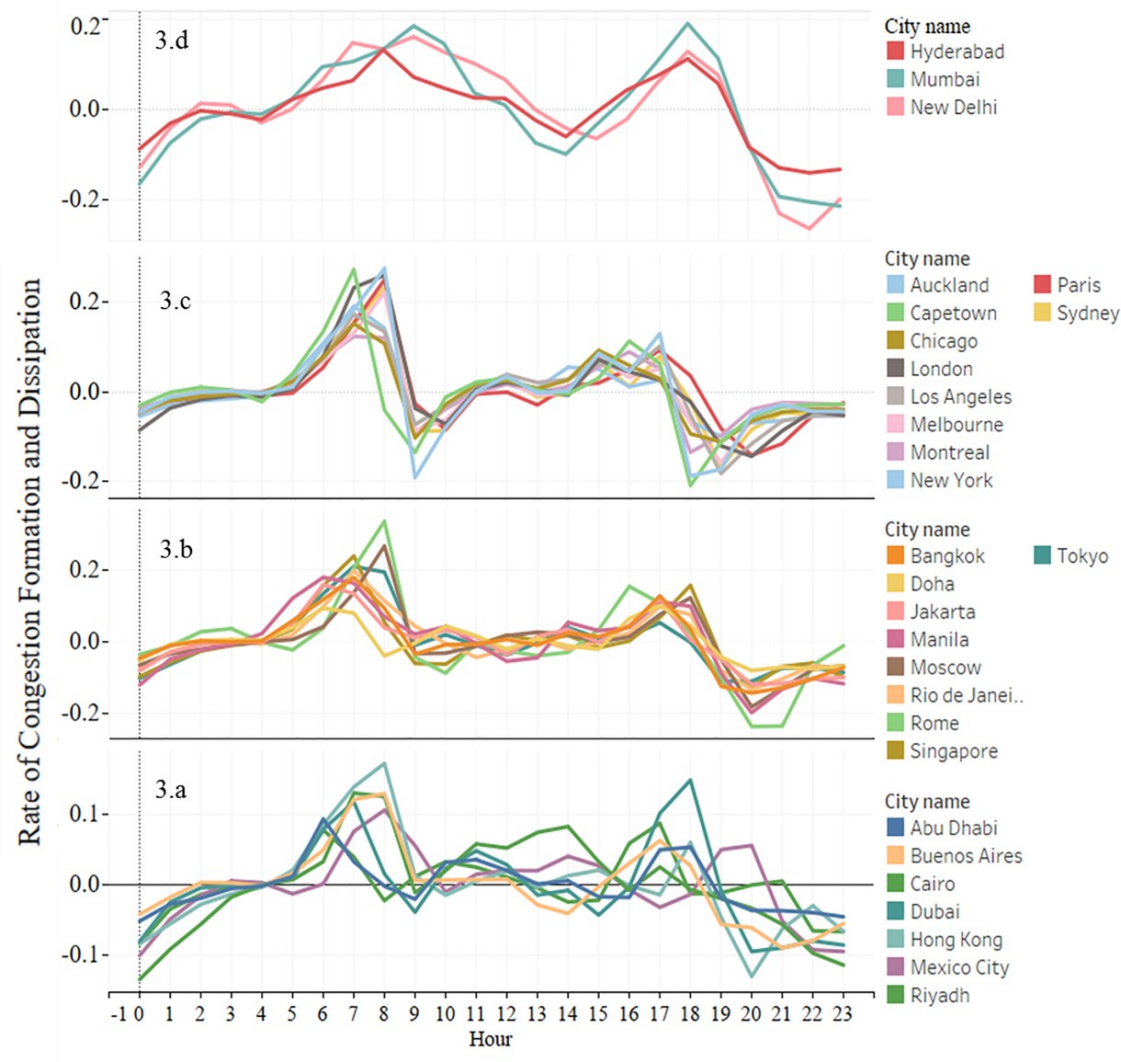
Formation and dissipation of congestion in different cities. **a.** cities with a low congestion index, **b. and c.** cities with moderate to high congestion index, **d.** an exception where the rate of dissipation is found to increase as the congestion increases.

The space-time evolution of congestion has also attracted great interest in recent years. Particularly, estimating the temporal evolution of congestion within a day, i.e. the rate of formation and dissipation of congestion aids in several strategic planning decisions. We estimate the rate of congestion formation (*θ*_1_) and dissipation (*θ*_2_) for each city as shown in Eq 2. Furthermore, we perform a k-means clustering based on the estimated *θ*_1_ and *θ*_2_ values (see Figs 3 and 4) to identify the cities with a similar rate of congestion formation and dissipation profiles.

The rate of congestion formation (*θ*_1_) and dissipation (*θ*_2_) for each city can be interpreted as the fractional congestion index increase and decrease experienced in one hour, respectively.

$$\theta_{t1},\theta_{t2}=\left|\frac{CI_{t}-CI_{t-1}}{\Delta t}\right|$$ (2)

*θ*_1_ and *θ*_2_ can be interpreted as the fractional congestion index increase and decrease experienced in one hour, respectively. A high index often prevails with hia gh rate of formation and low rate of dissipation.

Cluster (a) includes cities with a low congestion index. The congestion starts forming around 6AM in the morning and dissipates only around 12PM and the maximum rate of congestion formation (*θ*_1_ = 0.09 to 0.17) is comparatively low and dissipation (*θ*_2_ = 0.04 to 0.13) is comparatively high. This cluster mainly includes the Middle Eastern cities whose average speeds are high (Fig 1) and closer to the free flow speed (no distinct peak hour). These are also the cities with less population density. Most of these cities are highly developed with relatively newer road infrastructure with high capacity than the cities from other clusters. An exception within this cluster is Cairo which has a high congestion index.

Cluster (b) includes cities with moderate to high congestion index and they exhibit moderate to high *θ*_1_ and *θ*_2_ ranging from 0.1 to 0.33 and 0.3 to 0.12. However, the formation and dissipation patterns are similar to Cluster (a). This cluster predominantly includes developing cities of the Asian continent that are highly populated and show an extended peak (single peak) pattern.

Cluster (c) includes cities that have a proper morning and evening peak system that last for around 2 to 4 hours. Generally, *θ*_1_ (0.12 to 0.28) observed during the evening peak is higher and *θ*_2_ (0.03 to 0.08) is comparatively lower than during the morning peak. It includes most of the developed countries that have a moderate congestion index.

Finally, Cluster (d) is an exception where the rate of dissipation is found to increase as the congestion increases. It includes Indian cities that exhibit a high average congestion index, a moderate *θ*_1_ (0.13 to 0.18) and a high *θ*_2_ (0.08 to 0.16). They are highly populated metropolitan cities that exhibit a late morning peak and evening peak unlike cities from other clusters.

### 3.3 A comparison of day-to-day congestion profiles

**Fig 5:**
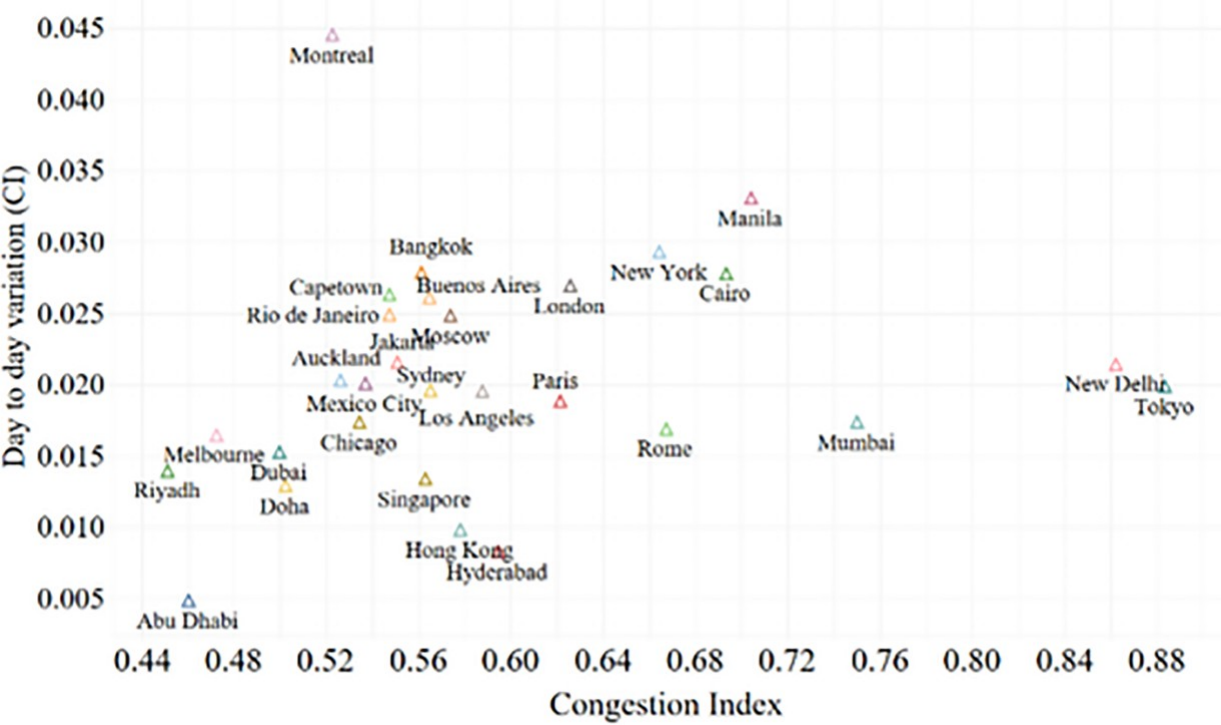
Reliability.

Travelers often plan their trips accommodating the expected regular delays in congestion and get frustrated by unexpected delays. The day-to-day variation in delay (or the congestion index, in this study) signifies the sensitivity of the city towards the congestion induced decline in reliability. A small value for the congestion index often prevails together with low variance and the variance in mean congestion increases as the congestion index increases. The theoretical relationship between mean congestion index and its standard deviation across days can be expressed by Eq 3, where *σ*′(*CI*) is the standard deviation of congestion index across multiple days, *μ*′(*δ*) is the mean value of the congestion index across multiple days $\gamma_{1}^{\prime },\;\gamma_{2}^{\prime }$ are coefficients and *ε*′ is the random error. A greater $\gamma_{2}^{\prime }$ indicates that the network is highly sensitive to a congestion induced decline in reliability. The $\gamma_{2}^{\prime }$ of all the cities except Montreal are less than 5% (see Fig 5). The comparatively higher $\gamma_{2}^{\prime }$ (0.085) for Montreal is possibly due to the winter snow storm during the data collection period [42].

$$\sigma^{\prime }\left(CI\right)=\gamma_{1}^{\prime }+\gamma_{2}^{\prime }\mu^{\prime }\left(CI\right)+\varepsilon^{\prime }$$ (3)

### 3.4 Spatial dispersion (heterogeneity) of congestion

**Fig 6:**
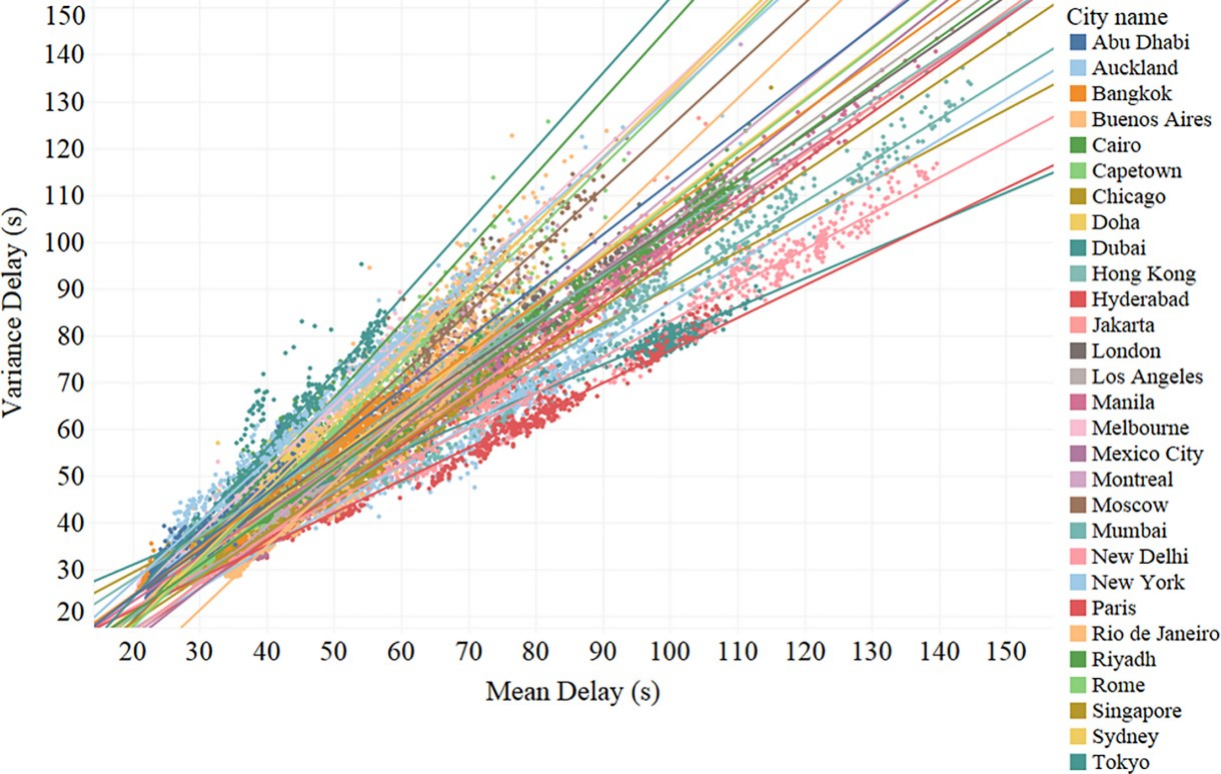
Rate of spatial dispersion of various cities. Mean delay of each link in the network (city) is fitted against the standard deviation of delay.

Congested traffic condition of a network can be characterized from the perspective of the rate of spatial dispersion. Spatial heterogeneity refers to the tendency for the distribution of geographical features or the process of geographical phenomena to be statistically non-stationary [43]. In other words, the traffic condition of road segments varies largely in different spatial locations; some are congested at a particular instant, whereas other road sections are probably not congested at the same instant. There are different measures to quantify the spatiotemporal heterogeneity of a road network. For example, Gao et. al (2016) used the metric, called the ratio of area, on vehicle speed data to capture heterogeneity [36]. The rate of spatial dispersion is modeled using the key statistics of the delay distribution: mean and standard deviation, with one depicting the central tendency and the other describing the dispersion (Fig 6). In general, the theoretical relationship between mean delay and its standard deviation can be expressed by Eq 4, where *σ*(*δ*) is the standard deviation of delay, *μ*(*δ*) is the mean value of the delay *γ*_1_, *γ*_2_ are coefficients and *ε* is the random error (see Table 2).

$$\sigma \left(\delta \right)=\gamma_{1}+\gamma_{2}\mu \left(\delta \right)+\varepsilon$$ (4)

**Table 2:** Spatial dispersion regression results.

City	*γ*_1_	*γ*_2_	*R*^2^
Auckland	1.17	1.3	0.98
Abu Dhabi	2.08	1.1	0.9
Bangkok	3.71	1.03	0.98
Mumbai	2.25	0.89	0.98
Cairo	0.06	1.02	0.99
Paris	-4.49	1.02	0.99
Jakarta	-3.71	1.02	0.95
Capetown	-10.59	1.41	0.97
New Delhi	6.39	0.77	0.98
Moscow	-7.66	1.32	0.97
Doha	-10.42	1.43	0.97
Dubai	-8.34	1.6	0.96
Buenos Aires	-19.77	1.37	0.96
Rome	-2.39	1.1	0.97
Rio de Janeiro	-7.27	1.39	0.97
Hong Kong	9.2	0.93	0.98
Hyderabad	7.3	0.69	0.98
New York	-0.22	0.87	0.94
Los Angeles	-0.52	1.04	0.96
London	4.28	0.99	0.98
Melbourne	-3.07	1.36	0.98
Mexico City	-8.07	1.13	0.95
Manila	3.8	0.96	0.99
Tokyo	18.67	0.61	0.97
Chicago	-0.76	0.96	0.97
Riyadh	-12.98	1.59	0.93
Singapore	14	0.76	0.98
Sydney	-0.92	1.1	0.97
Montreal	-8.21	1.18	0.94

**Fig 7:**
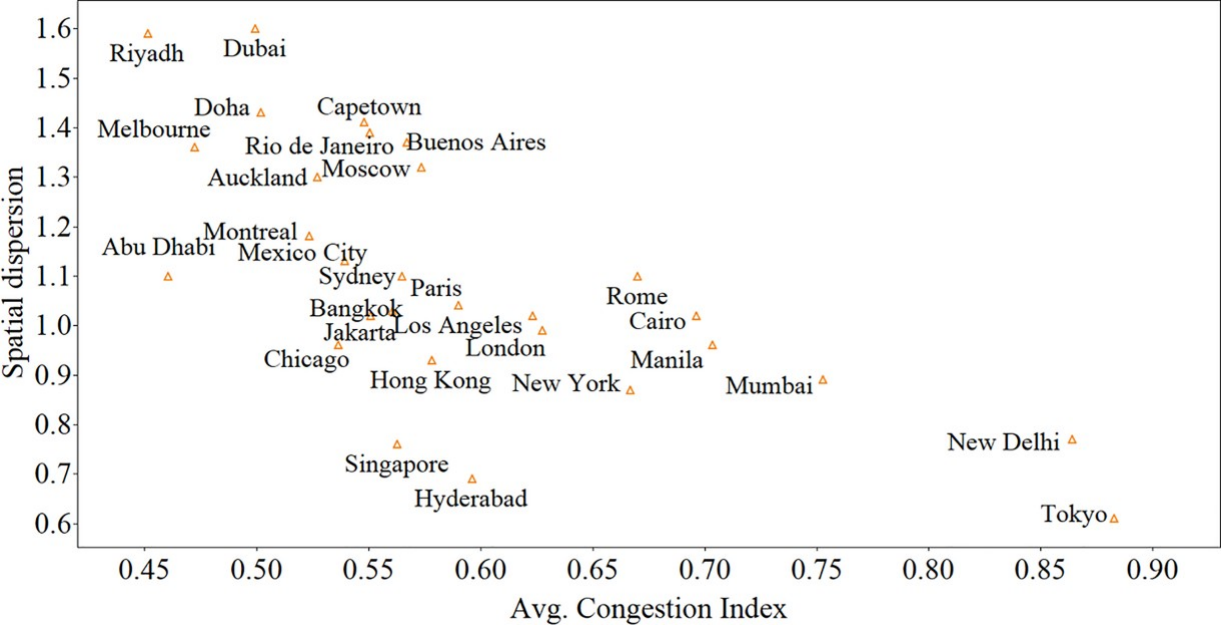
Rate of spatial dispersion of various cities plotted against the average CI of the city.

A small value for the delay which corresponds to the less congested traffic conditions often prevails together with low standard deviation. As congestion increases, the average speed of the network decreases due to the interaction of the vehicles, which causes a fluctuation in the speed and thus affect the average delay and the variation in delay increases. The magnitude of *γ*_2_ reflects the extent to which the standard deviation will increase with the increase in mean delay. A greater value of *γ*_2_ indicates that the network becomes more spatially heterogeneous as the mean delay increases and spatial dispersion is high during the peak hours in comparison to the off-peak hours. Fig 7 relates *γ*_2_ of a city with its average CI. The dispersion level (*γ*_2_) is different for different cities and has a negative correlation with the congestion index. That is, we find that the rate of spatial dispersion is lower in highly congested cities. In other words, congestion is not limited to certain areas of these cities, but widespread across the network.

### 3.5 Network stability

The notion of equilibrium in urban transportation networks stems from the dependence of link travel times on the link flows. The problem of interest here is how the users (at congested or free flow conditions) will be dispersed among the possible paths when every driver tries to minimize their own travel time. It is reasonable to assume that the travel time and thereby the speed of each link changes with the inflow and outflow of users and the travel time of several of the network’s paths change as the link flows change. Since each user can be expected to behave independently, a true equilibrium condition will be reached when no user can improve his travel time or perceived travel time by unilaterally changing routes.

**Fig 8:**
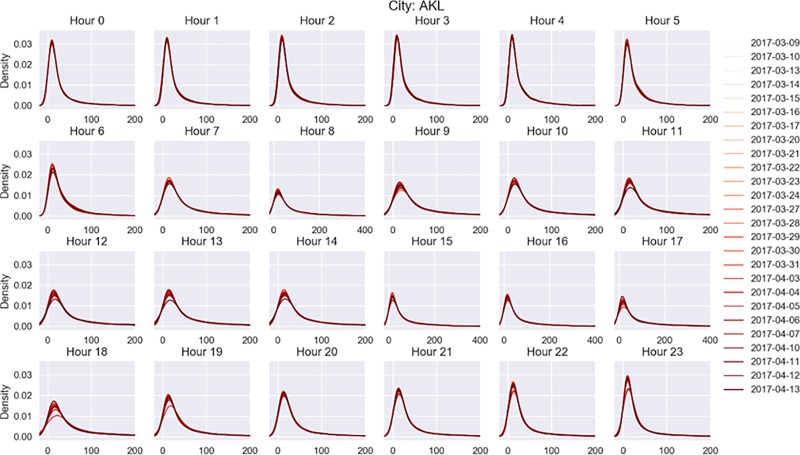
Stability of network equilibrium. Kernel density plot at every one hour for Auckland city that has the least overlap probability.

Network equilibrium has been the cornerstone of transport planning. Multiple equilibrium theories have been explored with mixed results [44]. However, irrespective of the type of equilibrium that exists they all predict a day-to-day stable distribution of speeds on links. The objective of this study is to empirically analyze the existence of a stable condition across days, during a given time period. We define the stability of network equilibrium by modeling its traffic characteristics as follows: At a given time *t* or a duration of time Δ*t*, the network equilibrium and the expected average delay of the network will be stable across days, if the distribution of delay across the network is the same. We obtain the delay profiles (delay distributions) of different cities at every hour for all the working days during the observation period and plot the kernel density estimates at every one hour to empirically analyse *the network stability* by examining the delay distribution plots. Inspection of the kernel density plots reveals that the distributions are homogeneous and stable across days for almost all the cities. An example (Auckland) is provided in Fig 8.

**Fig 9:**
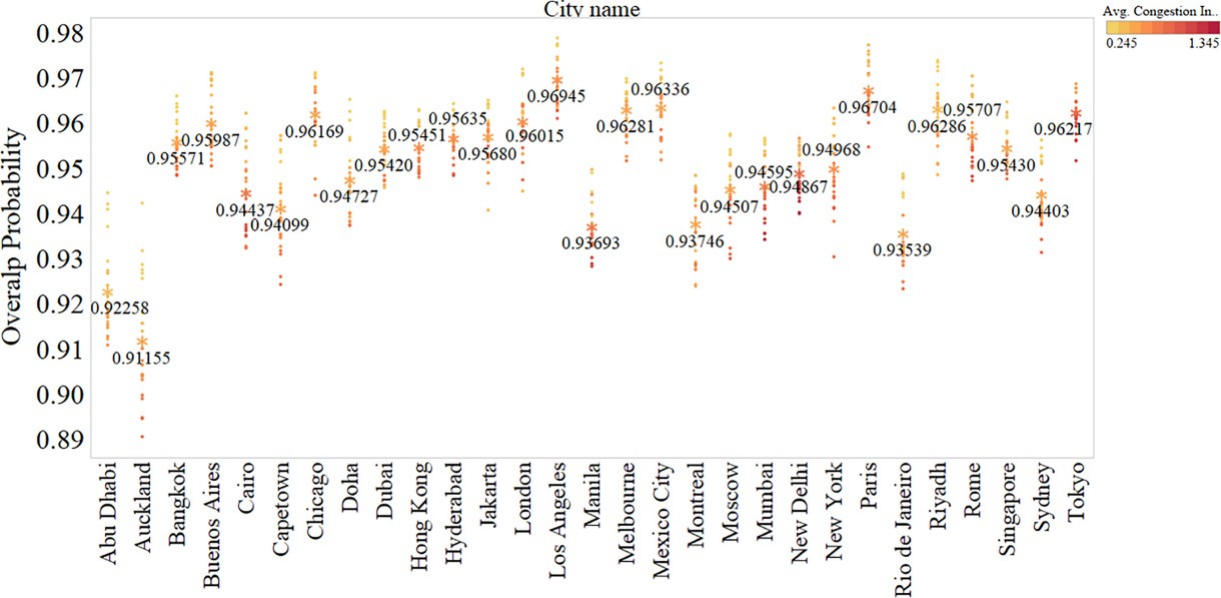
Stability of network equilibrium. Estimated overlap probability at every one hour for each city. The average estimated overlap probability values are highlighted and marked.

Further, to be able to compare and quantify the homogeneity and the spread of the delay distributions, we estimate the overlap probability for each pair of dates (Fig 9). An overlap probability value (*p*) closer to 1 indicates that the delay profiles are indifferent. That is, *at a given time t or a duration of time* Δ*t*, *the network equilibrium and the average expected delay of the network is stable across days*, *if the mismatch in probability (α) is minimal*.

Let the probability delay distribution plot for a duration of time Δ*t* and a given day *d* be represented by *n* speed bins *δ*_1_,*δ*_2_….*δ*_*n*_ having probabilities *p*(*δ*_1_),*p*(*δ*_2_)….*p*(*δ*_*n*_). According to the law of sum of probabilities $\sum_{i=1}^{n}p(\delta_{i})=1$. The expected delay $\bar{\delta_{\nabla t}^{d}}$ is given in Eq 5:

$$\hat{\delta_{\nabla t}^{d}}=\overset{n}{\underset{i=1}{\sum }}\delta_{i}.p\left(\delta_{i}\right)$$ (5)

Assume a new probability distribution for another day *d*′ which has an overlap probability of (1−*α*). The maximum deviation in the expected delay can be obtained by adjusting *α*/2 in the least and the highest delay bin. The new expected delay $\hat{\delta_{\nabla t}^{d^{\prime }}}$ and its relationship between $\bar{\delta_{\nabla t}^{d}}$ is given as follows: As $\alpha \to 0,\;\hat{\delta_{\nabla t}^{d^{\prime }}}=\hat{\delta_{\nabla t}^{d}}$

$$\hat{\delta_{\nabla t}^{d^{\prime }}}=\delta_{1}.\left\{p\left(\delta_{i}\right)-\frac{\alpha }{2}\right\}+\overset{n-1}{\underset{i=2}{\sum }}\delta_{i}.p\left(\delta_{i}\right)+\delta_{n}.\left\{p\left(\delta_{n}\right)+\frac{\alpha }{2}\right\}$$ (6)

$$\hat{\delta_{\nabla t}^{d^{\prime }}}=\hat{\delta_{\nabla t}^{d}}+\frac{\alpha }{2}.\left(\delta_{1}-\delta_{n}\right)$$ (7)

The average overlap probability values of each city are shown in Fig 9. The x-axis represents the number of road segments in the network; the y-axis represents the overlap probabilities of the delay distributions (from 00.00 to 24.00 at 1-hour interval). An overlap probability value closer to 1 indicates that the network is stable. From Fig 9, we can see that the distributions are strongly homogenous across days for all the cities with overlap probabilities greater than 0.935 except for Abu Dhabi and Auckland where the average overlap probabilities are 0.92 and 0.91 respectively.

In general, the overlap probabilities are observed to be higher during the off-peak (low demand) hours compared with the peak (high demand) hours. This finding is unsurprising because less traffic activity happens during the off-peak hours and hence traffic speed distribution across the city will be more stable. Furthermore, we found that the congested cities like New Delhi, Mumbai, Tokyo, Cairo, London, Paris, New York, and Rome are relatively more stable than the other cities.

## 4. Conclusion

Road traffic congestion has significant adverse impacts on the economy, environment, and society as a whole. Thus, measuring congestion and understanding its spatial and temporal patterns are critical to developing sustainable transport networks. In recent years, crowdsourced data has become increasingly popular among transport agencies as it provides easy accessibility to the way individuals travel in a road network. This study shows the utility of crowdsourced data to determine traffic conditions and, to the best of our knowledge, proposes a standard data source which facilitates comparison of traffic congestion across multiple cities of the world. This study proposed generalized congestion and stability metrics to compare and contrast speed variation patterns, congestion levels, its degree of formation and dissipation, spatial dispersion, and the stability of network equilibrium.

In this study, we defined a congestion index in terms of the proportion of travel time lost in congestion and showed that the congestion index of different cities is strongly related to emissions, population density, and GDP per capita. Then we showed the congestion formation and dissipation profiles of different cities and further clustered the cities with similar profiles. We then discussed the theoretical relationship between the mean spatial delay of the network and its standard deviation. We found that the standard deviation of delay, a measure of spatial heterogeneity, monotonically increases with the increase in the average delay and the heterogeneity level is different for different cities which has a negative correlation with their congestion index. We empirically analyzed the existence of a stable equilibrium condition in the network across days. We found that the distribution of speed and the expected average speed of the network is stable across days for a given time period and the stability increases as the number of segments in the network increases.
