# Correction: Characterizing multicity urban traffic conditions using crowdsourced data

**DOI:** 10.1371/journal.pone.0215728

**Published:** 2019-04-16

**Authors:** Divya Jayakumar Nair, Flavien Gilles, Sai Chand, Neeraj Saxena, Vinayak Dixit

There are errors in the Author Contributions. The correct contributions are: Conceptualization: DJN SC NS VD. Data curation: DJN FG SC NS. Formal Analysis: DJN FG SC NS. Investigation: DJN FG SC VD. Methodology: DJN SC VD. Resources: VD. Supervision: VD. Validation: DJN SC. Writing–original draft: DJN NS. Writing–review & editing: DJN SC NS.

## References

[1] Nair DJ, Gilles F, Chand S, Saxena N, Dixit V (2019) Characterizing multicity urban traffic conditions using crowdsourced data. PLoS ONE 14(3): e0212845 10.1371/journal.pone.0212845 30861011 PMC6413893

